# A case of rupture of right coronary sinus of Valsalva aneurysm after aortic root replacement due to Stanford type A aortic dissection 15 years ago

**DOI:** 10.1007/s12574-020-00505-6

**Published:** 2021-01-03

**Authors:** Satoshi Koto, Koichiro Imai, Ryotaro Yamada, Teruyoshi Kume, Yoji Neishi, Shiro Uemura

**Affiliations:** Department of Cardiology, Kawasaki Medical School, 577 Matsushima, Kurashiki, 701-0192 Japan

## Case report

**Fig. 1:**
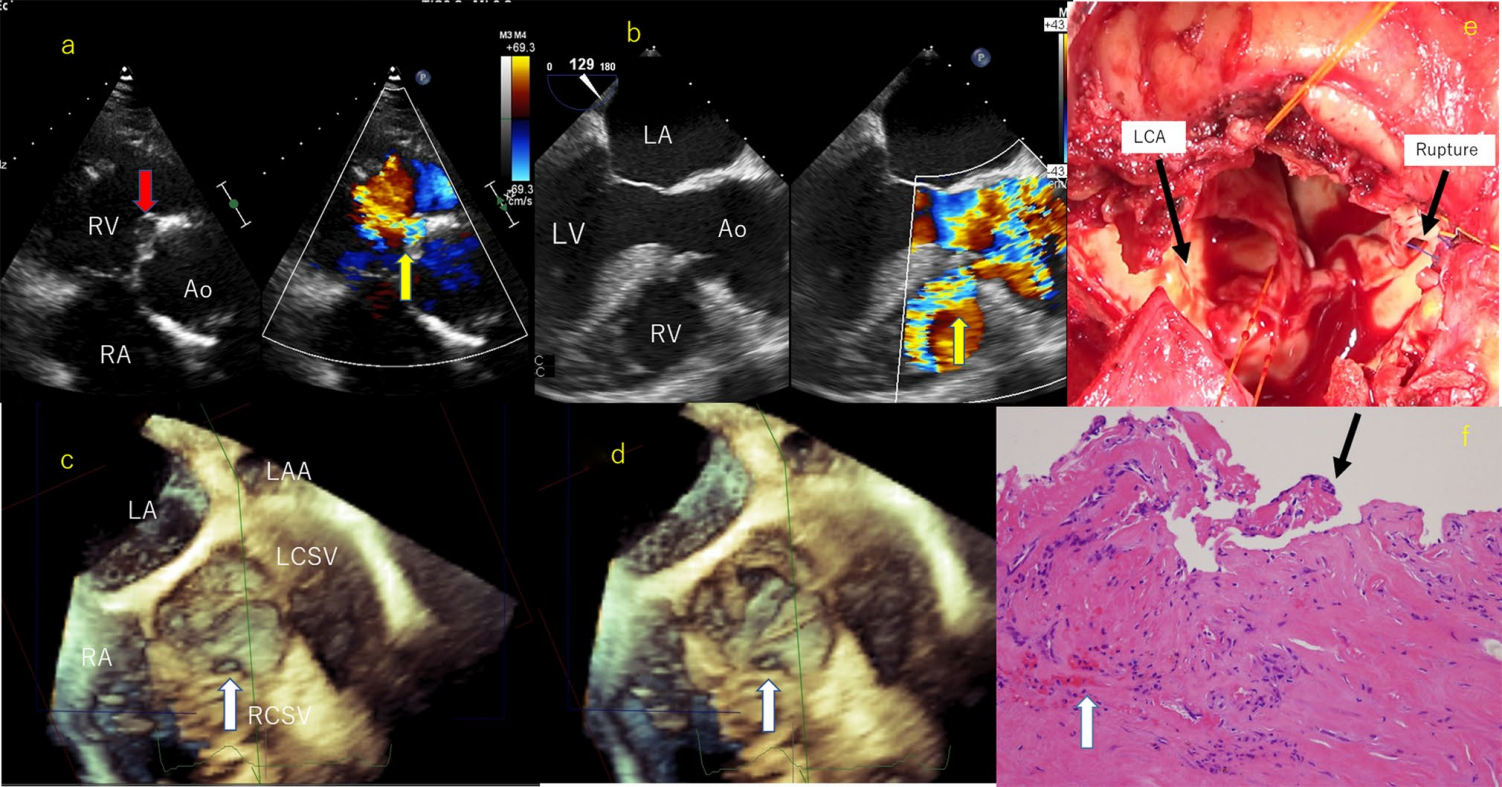
HYPERLINK "sps:id::fig1||locator::gr1||MediaObject::0"**a** Transthoracic echocardiograph by color Doppler shows right coronary sinus of Valsalva aneurysm (red arrow) and shunt through from right coronary sinus of Valsalva to right ventricle (yellow arrow). *RA* right atrium, *RV* right ventricle, *Ao* aorta. **b** Transesophageal echocardiography by color Doppler shows shunt through from right coronary sinus of Valsalva to right ventricle (yellow arrow). *LA* left atrium, *LV* left ventricle, *RV* right ventricle, *Ao* aorta. **c** Three-dimensional transesophageal echocardiography detect defect in right coronary sinus of Valsalva (white arrow) in diastole. *LA* left atrium, *RA* right atrium, *LCSV* left coronary sinus of Valsalva, *RCSV* right coronary sinus of Valsalva, *LAA* left atrial appendage. **d** Three-dimensional transesophageal echocardiography detect defect in right coronary sinus of Valsalva (white arrow) in systole. **e** Operative findings: view from the left ventricle side. Rupture of right coronary sinus of Valsalva. **f** Photomicrograph showing the inner wall of the excised artificial blood vessel. Fibrous capsule appears (black arrow) and fibrin deposition is also observed (white arrow) (hematoxylin and eosin stain, original magnification 3 × 20 × 2)

A 70-year-old Japanese man presented with symptom of a dyspnea and diagnosed as rupture of right coronary of sinus of Valsalva aneurysm. He had a history of ascending aortic replacement because of Stanford type A aortic dissection 15 years ago. Cardiovascular examination revealed a grade IV/VI continuous cardiac murmur at the right second intercostal space. Transthoracic echocardiography (TTE) revealed shunt through from right coronary sinus of Valsalva aneurysm to right ventricle detected by color Doppler (Fig. 1a) Two- and three-dimensional transesophageal echocardiography (3DTEE) revealed a ruptured aneurysm of the right coronary sinus of Valsalva causing a large left-to-right shunt through into the right ventricle (Fig. 1b) and defect in right coronary sinus of Valsalva during cardiac cycle (Fig. 1c, d). We decided to perform re-ascending aorta replacement with aortic valve replacement. A ruptured coronary aneurysm was observed in the right sinus of Valsalva. The rupture site was accompanied by the destruction of the wall structure (Fig. 1e).

Photomicrograph showing the inner wall of the excised artificial blood vessel. Fibrous capsule appears (black arrow) and fibrin deposition is also observed (white arrow) (hematoxylin and eosin stain, original magnification 3 × 20 × 2) (Fig. 1f).

We experienced a case of ruptured sinus of Valsalva aneurysm after ascending aortic replacement for Stanford type A dissection 15 years ago. In this case, gelatin–resorcin–formalin (GRF) glue was used for ascending aortic replacement. The surgical performance of acute aortic dissection had greatly improved by the appearance of GRF glue since 1980. However, stump-plasty using GRF glue has been reported to cause postoperative anastomotic complications in 5–20% [1, 2]. Pathological examination showed fragility of the aorta media and coagulation necrosis due to formalin-induced tissue injury [3]. Although GRF glue is rarely used, it had been used in many cases previously. Therefore, it is necessary to observe the occurrence of anatomic complications carefully over the long term.

## References

[1] Hata M, Shiono M, Sezai A (2004). Type A acute aortic dissection: immediate and mid-term results of emergency aortic replacement with the aid of gelatin resorcin formalin glue. Ann Thorac Surg.

[2] Izutani H, Shibukawa T, Kawamoto J (2007). Devastating late complication for repair of type A acute aortic dissection with usage of gelatin-resorcinol-formalin glue. Int Cardiovasc Surg.

[3] Kazui T, Washiyama N, Bashar AH (2001). Role of biologic glue repair of proximal aortic dissection in the development of early and midterm redissection of the aortic root. Ann Thorac Surg.

